# Blood Microbiome Quantity and the Hyperdynamic Circulation in Decompensated Cirrhotic Patients

**DOI:** 10.1371/journal.pone.0169310

**Published:** 2017-02-01

**Authors:** Daniela Traykova, Beacher Schneider, Mario Chojkier, Martina Buck

**Affiliations:** 1 Department of Medicine, University of California, San Diego, La Jolla, CA, United States of America; 2 Veterans Affairs San Diego Healthcare System, San Diego, CA, United States of America; 3 Biomedical Sciences Program, University of California, San Diego, La Jolla, CA, United States of America; 4 Clinical Translational Research Institute, University of California, San Diego, La Jolla, CA, United States of America; Taipei Veterans General Hospital, TAIWAN

## Abstract

**Background:**

Recently, a complex microbiome was comprehensibly characterized in the serum and ascitic fluid of cirrhotic patients. In the current study, we investigated for the first time the induction of inflammatory pathways and Nitric Oxide, as well as the systemic hemodynamics in conjunction with the blood microbiome in a Child-Pugh class B cirrhotic cohort.

**Methods and Findings:**

We used the Intestinal Infections Microbial DNA qPCR Array to screen for 53 bacterial DNA from the gut in the blood. Assays were designed using the 16S rRNA gene as a target, and PCR amplification primers (based on the Human Microbiome Project) and hydrolysis-probe detection. Eighteen systemic hemodynamic parameters were measured non-invasively by impedance cardiography using the BioZ ICG monitor. The inflammatory response was assessed by measuring blood cytokines, Nitric Oxide RNA arrays, and Nitric Oxide. In the blood of this cirrhotic cohort, we detected 19 of 53 bacterial species tested. The number of bacterial species was markedly increased in the blood of cirrhotic patients compared to control individuals (0.2+/-0.4 vs 3.1+/-2.3; 95% CI: 1.3 to 4.9; P = 0.0030). The total bacterial DNA was also increased in the blood of cirrhotic subjects compared to control subjects (0.2+/- 1.1 vs 41.8+/-132.1; 95% CI: 6.0 to 77.2; P = 0.0022). In the cirrhotic cohort, the Cardiac Output increased by 37% and the Systemic Vascular Resistance decreased by 40% (P< 0.00001 for both compared to control subjects). Systemic Vascular Resistance was inversely correlated to blood bacterial DNA quantity (- 0.621; 95% CI -0.843 to -0.218; P = 0.0060), blood bacterial species number (- 0.593; 95% CI -0.83 to -0.175; P = 0.0095; logistic regression: Chi Square = 5.8877; P = 0.0152), and serum Nitric Oxide (- 0.705; 95% CI -0.881 to -0.355; P = 0.0011). Many members of the Nitric Oxide signaling pathway gene family were increased in cirrhotic subjects.

**Conclusions:**

Our study identified blood bacterial DNA in ~ 90% of the cirrhotic patients without clinical evidences of infection, and suggests that the quantity of bacterial DNA in blood may stimulate signaling pathways, including Nitric Oxide, that could decrease systemic vascular resistance and increase cardiac output.

## Introduction

Inflammation contributes to the pathogenesis of experimental liver disease [1],[2],[3],[4],[5],[6] and it is associated with most liver diseases in patients [7]. Inflammasomes are multiprotein complexes that can sense danger signals from pathogens and assemble to mediate caspase-1 activation, which proteolytically activates the pro-inflammatory cytokines IL-1β and IL-18 [7].

Activated pro-inflammatory macrophages and vascular endothelial cells express Nitric Oxide Synthetase (NOS2) and produce Nitric Oxide (NO) [8],[9], which induces vasodilation and decreases Systemic Vascular Resistance (SVR) in both bacterial sepsis [10],[11],[12] and in cirrhosis [13], [14], [15]. Bacterial liposaccharide (LPS) and bacterial DNA are danger signals that can activate macrophages through Toll-like receptors 4 and 9, respectively [16],[17]. The clinically unapparent translocation of bacteria, bacterial LPS and bacterial DNA to the mesenteric lymph nodes, blood and ascitic fluid occurs in patients with cirrhosis [18], probably mimicking some of the inflammatory and hemodynamic responses to sepsis.

Cirrhotic patients have a hyperdynamic systemic circulation, characterized by a decrease in SVR and an increase in Cardiac Output (CO) [15],[19]. The systemic hyperdynamic circulation is a major contributor to the morbidity and mortality among cirrhotic patients [20]. Increased hepatic vein pressure gradient (HVPG) is an indicator of liver sinusoidal pressure as well as of the severity of the liver disease [21], and it is linked to the hyperdynamic circulation of cirrhosis [15]. Increased HVPG was associated with the presence of serum bacterial DNA after a meal, suggesting a possible causal effect between postprandial bacterial translocation and an increase in HVPG [22].

However, previous studies assessing inflammatory biomarkers, bacterial LPS or bacterial DNA [22],[23],[24], [25], [26] have not determined either the bacterial species or the bacterial DNA quantity translocated in cirrhotic patients. Recently, Santiago et al [27] have comprehensibly characterized a complex microbiome in the serum and ascitic fluid of cirrhotic patients, but the aims of these investigators did not include correlative analysis of inflammatory pathways or the systemic hyperdynamic circulation of cirrhosis.

To date no concurrent determination of the blood microbiome and the signaling pathways resulting in the systemic hyperdynamic circulation has been measured in a cirrhotic cohort.

Therefore, we utilized the Intestinal Infections Microbial DNA qPCR Array representing 5 phyla, 12 bacterial orders and 53 bacterial species from the intestinal pathogen microbiome to detect their presence in blood, as well as to measure 18 systemic hemodynamic parameters by non-invasive impedance plethysmography, and the inflammatory response by measuring cytokines, NO RNA arrays, NO, and LBP in cirrhotic patients with a history of clinically significant portal hypertension.

Our study provides the first correlative analysis of the blood microbiome, inflammatory biomarkers and systemic hemodynamics in a cirrhotic cohort.

## Methods

### Participants

Written informed consent was obtained from each patient included in the study before any procedures were performed, and the study protocol conforms to the ethical guidelines of the 1975 Declaration of Helsinki as reflected in an approval by the institution's human research committee before the study started. The protocol was approved by the Veterans Affairs San Diego Healthcare System.

Eligible patients were enrolled from the Liver Clinic if they met all of the inclusion and none of the exclusion criteria. They had cirrhosis and evidences of ascites, esophageal-gastric varices or hepatic encephalopathy, and were older than 18 and less than 75 years of age. Exclusion criteria included hepatocellular carcinoma, splenic, mesenteric or portal vein thrombosis, treatment with β-blockers or portal-systemic shunting procedures, treatment of arterial hypertension, pregnancy, and excessive alcohol intake (> 2 drinks per day) during the preceding 3 months. Additional exclusion criteria for the cirrhotic patients included liver transplant, hemoglobin <10 g/dL, a Cockcroft-Gault glomerular filtration rate (GFR) < 60 mL/minute, arterial hypertension, and type 2 diabetes. Exclusionary criteria were also the use of an investigational drug, systemic immunosuppressants (including corticosteroids), immunomodulation agents, use of marijuana or an infection within 4 weeks before the screening visit, use of illicit drugs, history of pulmonary or cardiac disease, uncontrolled thyroid disease, or hematologic disorders. Control subjects were healthy individuals without liver disease. Our study report follows the STROBE checklist and some pertinent STROME-ID checklist items.

### Specific Intestinal Microbiome Array System

We used the Intestinal Infections Microbial DNA qPCR Array (Qiagen, Cat #BAID-140 3Z) to screen the blood (plasma) for pathogenic bacteria from the gut. Assays were designed using the 16S rRNA gene as a target, and PCR amplification primers (based on the Human Microbiome Project) and hydrolysis-probe detection, which increases the specificity and sensitivity of each assay. To maintain a working environment free of DNA contamination we physically separated the workspaces used for PCR setup and post-PCR processing operations. We decontaminated PCR workspace and labware (pipets, tube racks, etc.) with UV light before each new use to render any contaminated DNA ineffective in PCR through the formation of thymidine dimers or with 10% bleach to chemically inactivate and degrade any DNA. We did not open any previously run and stored PCR array plate. We carefully removed the thin-wall 8-cap strips or the adhesive film from PCR arrays since it could release PCR product DNA into the air where it can contaminate the results of future experiments.

Each assay was based on PCR amplification of a species-specific genetic region of the relevant bacteria using 500 ng genomic DNA. Purification of microbial genomic DNA was performed with the QI Amp (Qiagen #50112), and DNA dilution was performed with DNase-free water. We used triplicate plasma samples for each subject. The amplified product was detected using target-specific fluorescent hydrolysis probes. Assays for detection of bacterial species target the 16s rRNA gene and were designed using the Green-Genes database for 16s rRNA sequences. Pan-bacteria assays that detect a broad range of bacterial species served as positive controls in each subject’s plasma to test for the presence of inhibitors in the sample or the efficiency of the polymerase chain reaction itself using a pre-dispensed artificial DNA sequence and the primer set that detects it. Similarly, negative controls (Microbial DNA-Free Water) were used in each subject’s plasma to monitor for potential contamination (false positive results). Host GAPDH assays were used to detect the presence of human genomic DNA and to normalize for relative microbial quantification. This array contains a panel of proprietary controls to monitor DNA contamination as well as the first strand synthesis and real-time PCR efficiency. The specificity of each gene amplification is guaranteed by the RT^2^ SYBR Green PCR Array System (Qiagen, Cat #330502). Blood separation was performed with Histopaque (Sigma, Cat #1077). In brief, for each triplicate sample four separate PCR reactions were set up. These included controls for Positive PCR Control, No Template Control, Microbial DNA Positive Control and Microbial DNA qPCR Assay. Real-time PCR was performed on a Bio-Rad CFx96 with a setting of 40 cycles. Data was analyzed using web-based Qiagen analysis software.

### Systemic Hemodynamics

The systemic hemodynamic parameters were measured by non-invasive impedance plethysmography using the BioZ ICG monitor (BioZ; Cardio Dynamics, San Diego, CA) [28]. Eighteen systemic hemodynamic parameters were calculated or derived after subjects had been fasting for >4 hr., and in the recumbent position in a quiet room, and in the dark for 5 min.

### Cytokines/Chemokines

We used a Multi-Analyte ELISA Array kit (MEH-008A0; Qiagen) for the detection of cytokines/chemokines induced by microbial infection (tumor necrosis factor [TNF]-α, interleukin [IL]-1β, IL-6, IL12, IL-17A, IL-8, monocyte chemoattractant protein [MCP]-1, regulated on activation, normal T cell expressed and secreted [RANTES], macrophage inflammatory protein [MIP]-1α, MIP-1β, macrophage-derived chemokine [MDC] and Eotaxin) [6], [29].

### NO Gene Array

We used the RT^2^ Profiler™ PCR Array Human Nitric Oxide Signaling Pathway (Qiagen) to determine whether this pathway is activated by the bacterial DNA translocation in the cirrhotic subjects when compared to control subjects. This array contains a panel of proprietary controls to monitor genomic DNA contamination as well as the first strand synthesis and real-time PCR efficiency. The specificity of each gene amplification is guaranteed by the RT^2^ SYBR Green PCR Array System (Qiagen).

### NO Measurement

We used the Nitric Oxide Assay Kit (colorimetric) (ab 65328), which provides an accurate, convenient measure of total nitrate/nitrite in a simple two-step process. The first step converts nitrate to nitrite utilizing nitrate reductase. The second step uses Griess Reagents to convert nitrite to a deep purple azo compound. The amount of the azo-chromophore accurately reflects nitric oxide amount in samples. The detection limit of the assay is approximately 1 nmol nitrite/well.

### NALP-3 Analysis

Immunoprecipitation of NALP-3 from blood macrophages was performed in buffy-coat lysates using a NALP-3 specific antibody. The immunoprecipitated NALP-3 was quantified in an immunoblot, using β-actin for internal correction [6].

### LPB Analysis

An enzyme-linked immunoassay kit was used to determine plasma LPS-binding protein (LPB) according to the manufacturer’s protocol (Biomatrix).

### Blinding

Individuals performing the laboratory tests were kept blinded to the subjects’ demographics, clinical and hemodynamic data.

### Study Size

We initiated our study before the study by Santiago et al was published [27]. Therefore, there were no data available on the prevalence of intestinal microbiome translocation using a sensitive and comprehensive array. However, we considered a translocation rate of 25% in the blood microbiome to be relevant as proof of principle of the diversity of the bacterial translocation. To detect a change of 25% in bacterial translocation or in systemic hemodynamic parameters in the cirrhotic group assuming a standard deviation of 20% with a two-sided 5% significance level and a power of 90%, a sample size of 9 patients per group completing the study was optimal (*avoiding unnecessary enrollment of additional patients as recommended by the ethical guidelines for clinical research)*, and it was decided to enroll 9 subjects per group given the lack of dropout for a single visit.

### Statistical Analysis

Results are expressed as mean (+/-SD and 95% CI). Either the Student-*t* or the Wilcoxon Mann-Whitney tests were used to evaluate the differences of the means between groups, with a *P* value of <0.05 as significant. Wald’s 95% CI of the difference were calculated. Spearman’s correlation was also performed and 95% CI were calculated based on the Fisher r-to-z transformation. Linear and logistic regression analyses were also calculated when appropriate. All statistical analyses were performed using the Analyse-it program (www.Analyze-it.com).

## Results

### Demographic and Clinical Characteristics of the Subjects

Both groups consisted of 9 male subjects of a similar age (**Table 1**). The optimal sample size was determined as described above. The enrollment, allocation and analysis are depicted in **S1 Table**.

**Table 1:** Demographic and clinical data. The subjects’ demographics and clinical parameters are shown for the control and cirrhotic cohorts. The P values and the 95% CI are indicated for each comparison using a two-tailed Mann-Whitney U test.

Parameter	Control (mean +/- SD)	Cirrhotic (mean +/- SD)	P value (95% CI of difference)
Number	9	9	NS
Gender (male)	9	9	NS
Age (age)	60.6 +/- 5.9	62.0 +/- 6.1	0.9282 (-6.9)
AST (IU/ml)	24.6 +/- 7.3	41.1 +/- 14.5	0.0151(-27.1)
ALT (IU/ml)	21.0 +/- 5.8	26.4 +/- 13.0	0.5352(-14.7)
Albumin (g/dL)	4.54 +/- 0.28	2.92 +/- 0.60	0.0004(1.2)
Total bilirubin (mg/dL)	0.53 +/- 0.21	2.08 +/- 0.75	0.0004(-2.1)
Creatinine (mg/dL)	0.99 +/- 0.22	1.18 +/- 0.45	0.2891(-0.5)
INR (ratio)	1.07 +/- 0.10	1.40 +/- 0.24	0.0030(-0.5)
WBC (x 10^3^/μL)	6.66 +/- 1.94	6.32 +/- 3.87	0.8211 (-2.5)
Hgb (g/dL)	14.13 +/- 1.27	12.40 +/- 1.60	0.0223 (0.4)
Platelets (x 10^3^/μL)	232.4 +/- 46.2	96.3 +/- 56.4	0.0014(88.5)
ESR (mm/hr)	14.7 +/- 4.7	24.5 +/- 20.2	0.2218 (-23.3)
CRP (mg/dL)	0.17 +/- 0.18	1.33 +/- 1.36	0.0046 (-2.1)
Hgb A1c (%)	5.21 +/- 0.57	4.52 +/- 0.40	0.0173 (0.2)
MELD score	N/A	14.2 +/- 3.7	N/A
Child-Pugh score	N/A	9.56 +/- 1.24	N/A

The cirrhotic subjects had a statistically significant difference in aspartate aminotransferase (AST), albumin, total bilirubin, international normalized ratio (INR), hemoglobin, platelets, C-reactive protein (CRP), and hemoglobin A1C (**Table 1**). There were no statistically significant differences in alanine aminotransferase (ALT), creatinine, and erythrocyte sedimentation rate (ESR). The cirrhotic subjects had a Model for End-Stage Liver Disease (MELD) score of 14.2</-3.7 and Child-Pugh score of 9.56+/-1.24 (**Table 1**). All the cirrhotic subjects had ascites on enrollment (N = 5) or had previously had ascites (N = 4). The etiology of the cirrhosis was secondary to chronic hepatitis C infection in 7 subjects, with 5 having active hepatitis C infection at the time of the study (2 treatment naïve and 3 treatment experienced), while 2 had achieved sustained virological response to treatment. Two subjects had alcoholic cirrhosis.

### Hemodynamic Characteristics of the Subjects

Systemic hemodynamic parameters were measured non-invasively by impedance cardiography using the BioZ ICG monitor (BioZ; Cardio Dynamics, San Diego, CA). This technique has been extensively validated against the standard of care thermodilution technique (TD), and demonstrates a superior intrapatient reproducibility compared to Cardiac Output (CO)-TD measurements [28]. Eighteen systemic hemodynamic parameters were calculated or derived after subjects had been in the recumbent position in a quiet room, and in the dark for 5 min, and fasting for > 4 hrs. As expected, the cirrhotic subjects had evidences of a marked hyperdynamic circulation compared to control subjects with statistically significant differences in systolic blood pressure (SBP), diastolic blood pressure (DBP), mean arterial pressure (MAP), CO, cardiac index (CI), SVR, systemic vascular resistance index (SVRI), and stroke volume (SV) (**Table 2**). In the cirrhotic cohort, the CO increased by 37%and SVR decreased by 40% (P<0.00001 for both compared to control subjects). The differences between the groups in cardiac rate (CR), stroke volume index (SVI), velocity index (VI), acceleration index (ACI), thoracic fluid content (TFC), pre-ejection period (PEP), left ventricular ejection fraction (LVET), systolic time ratio (STR), left cardiac work (LCW), and left cardiac work index (LCWI) were not statistically significant (**Table 2**).

**Table 2:** Subjects’ hemodynamic parameters. The subjects’ systemic hemodynamic parameters are shown for the control and cirrhotic cohorts. The P values and the 95% CI are indicated for each comparison using a two-tailed Mann-Whitney U test.

Parameter	Control (mean +/- SD)	Cirrhotic (mean +/- SD)	P value (95% CI of difference)
Number	9	9	N/A
CR (beats/min)	65+/-7.2	71+/-9.9	0.0929 (-14.0)
SBP (mmHg)	136+/-17	115+/-12	0.0135 (7.4)
DBP (mmHg)	79+/-8	64+/-6	0.0030 (8.5)
MAP (mmHg)	98 +/-12	82+/-7	0.0093 (6.9)
CO (L/min)	5.48+/-1.3	7.50+/-1.4	0.0104 (-3.3)
CI (L/min/m^2^)	2.79+/-0.7	3.51+/-0.5	0.0192 (-1.3)
SVR (dynes x sec/cm^5^)	1374+/-228	830+/-133	0.0005 (371.6)
SVRI (dynes x sec/cm^5^ /m^2^)	2712+/-451	1753+/-222	0.0005 (630.6)
SV (mL)	87.33+/-17	105.56+/-15	0.0271 (-33.0)
SVI (mL/m^2^)	44.11+/-9	49.67+/-6	0.0929 (-12.6)
VI (x1000/sec)	42.89+/-16	51.67+/-18	0.2501(-24.5)
ACI (x100xsec^2^)	70.00+/-24	87.33+/-26	0.0929 (-40.4)
TFC (kohm)	36.33+/-5	41.37+/-7	0.1211 (-10.7)
PEP (msec)	117.56+/-21	109.00+/-23	0.6241 (-11.8)
LVET (msec)	309.11+/-35	303.78+/-25	0.7948 (-22.8)
STR (ratio)	0.39+/-0.1	0.36+/-0.1	0.6599 (-0.1)
LCW (kg x m)	7.06+/-2	7.83+/-2	0.3788 (-2.6)
LCWI (kg x m/m^2^)	3.59+/-1	3.67+/-1	0.4777 (-1.0)

As expected, the SVR had a statistically significant relationship with CO (- 0.873; 95%CI -0.951 to -0.686; P = 0.000002), CI (- 0.769; 95% CI -0.909 to -0.472; P = 0.0002) and MAP (0.524; 95% CI 0.076 to 0.796; P = 0.0257) (**Table 3**). SVR was correlated to bacterial DNA quantity (-0.621; 95% CI -0.843 to -0.218; P = 0.0060) and bacterial species number (- 0.593; 95%CI -0.83 to -0.175; P = 0.0095; logistic regression: Chi Square = 5.8877; P = 0.0152) (**Table 3**), suggesting but not proving the relevance of both translocated bacterial DNA quantity and bacterial species number on the abnormal SVR of cirrhosis.

**Table 3:** Correlation of Systemic Vascular Resistance with hemodynamic, laboratory, and bacterial parameters. The correlation of the Systemic Vascular Resistance with hemodynamic, laboratory and bacterial parameters are shown in the cirrhotic cohort. The P values and 95% CI are indicated for each comparison using the Spearman’s correlation.

Parameter	Spearman’s Correlation	P value (95% CI)
CO (L/min)	-0.873	0.000002 (-0.951 to -0.686)
CI (L/min/m^2^)	-0.769	0.0002 (-0.909 to -0.472)
MAP (mmHg)	0.524	0.0257 (0.076 to 0.796)
Albumin (g/dL)	0.784	0.0001 (0.51 to 0.917)
Total Bilirubin (mg/dL)	-0.694	0.0014 (-0.876 to -0.336)
INR (ratio)	-0.699	0.0013 (-0.879 to -0.345)
Platelets (x 10^3^/μL)	0.639	0.0043 (0.246 to 0.851)
CRP (mg/dL)	-0.689	0.0002 (-0.874 to -0.328)
Hgb A1c (%)	-0.518	0.0278 (-0.793 to -0.068)
Bacterial species (number)	-0.593	0.0095 (-0.83 to -0.175)
Bacterial species (DNA quantity)	-0.621	0.0060 (-0.843 to -0.218)
NO (μM)	-0.705	0.0011 (-0.881 to -0.355)

Given that advanced cirrhosis in patients and animals is associated with decreased SVR, it is congruent that SVR also had statistically significant associations with laboratory indicators of liver cirrhosis, including albumin, total bilirubin, INR, and platelets (**Table 3**). Cirrhotic decompensation is often related pathogenically to inflammation [26]. Indeed, in our cirrhotic cohort, this phenomenon was suggested by the high negative relationship between SVR and CRP (- 0,689; -0.874 to -0.328; P = 0.0002) (**Table 3**). Thus, CRP appears to reflect the inflammatory cascade probably induced or contributed to by bacterial translocation resulting in decreased SVR in cirrhosis.

### Intestinal Microbiome in Blood of the Subjects

**Fig 1:**
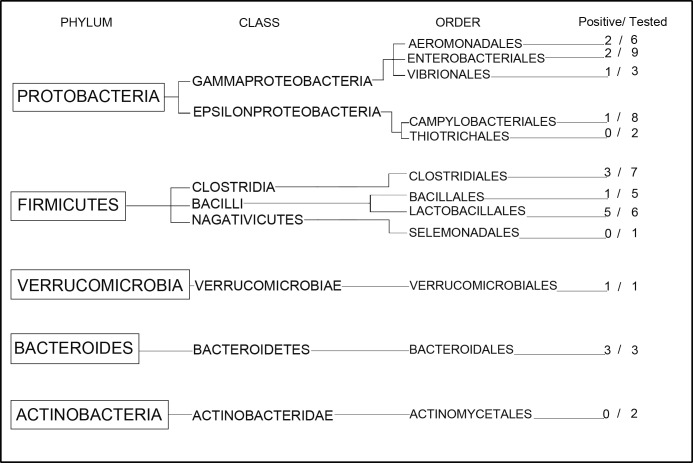
The bacterial DNA phylum, class, and order detected in the blood of cirrhotic subjects. Four phyla were detected in the blood of cirrhotic patients: Firmicutes, Protobacteria, Bacteroides and Verrucomicrobia. In this cirrhotic cohort we detected 19 of 53 bacteria species tested (Positive/Tested).

**Fig 2:**
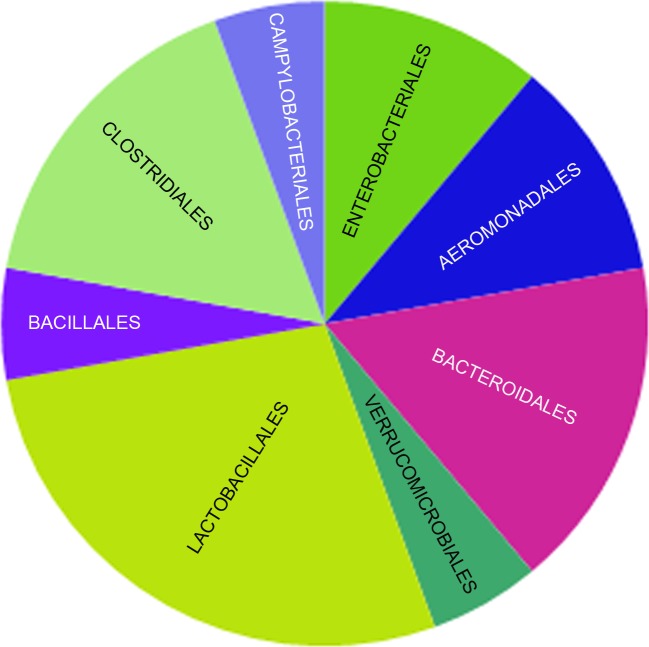
The bacterial DNA orders detected in the blood of cirrhotic subjects. We found 9 out of 12 bacterial orders in cirrhotic subjects.

We were able to detect bacteria from 4 of the 5 phyla tested (Protobacteria, Firmicutes, Verrucomicrobia, and Bacteroides) in the blood of cirrhotic subjects, (**Fig 1)**. Blood of the cirrhotic subjects contained 6 of 8 bacteria classes (Gammaproteobacteria, Epsilonproteobacteria, Clostridia, Bacilli, Verrucomicrobiae, and Bacteroidetes) (**Figs 1 and 2**). Bacteria classes Negativicutes and Actinobacteridae were not detected (**S2 Table)**.

We found 9 bacterial orders in cirrhotic subjects (Aeromonadales [2 bacteria species], Enterobacteriales [2 bacteria species], Vibrionales (1 bacterium specie), Campylobacteriales [1 bacterium specie], Clostridales [3 bacteria species], Bacillales [1 bacterium specie], Lactobacillales [5 bacteria species], Verrucomicrobiales [1 bacterium specie], and Bacteroidales [3 bacteria species]) (**Figs 1 and 2 and Table 4)**.

**Table 4:** Blood bacteria DNA identified in control and cirrhotic subjects. The blood bacterial DNA phylum, class, order, and species identified are shown for the control and cirrhotic cohorts. Bacterial species’ DNA quantity per subject and total are shown for the two cohorts. The P values and the 95% CI are indicated for each comparison using a two-tailed Mann-Whitney U test.

Blood Bacteria (Taxonomic #)	Control (mean +/- SD)	Cirrhotic (mean +/- SD)	Phylum
			*Class*
			Order
Bacterial species (number)/subject	0.2+/-0.4	3.1+/-2.3 (95% CI: 1.3 to 4.9; P = 0.0030)	
Bacterial species (DNA quantity)/subject	0.2+/-1.1	41.8+/-132.1 (95% CI: 6.0 to 77.2; P = 0.0022)	
Bacterial orders/cohort	2/12	9/12 (P = 0.0041)	
Aeromonas veronii (959036),		4.3	**Protobacteria**
			*Gammaproteobacteria*
Aeromonas sobria (959035)			Aeromonadales
Akkermansia muciniphila (959114)		11.4	**Verrucomicrobia**
			*Verrucomicrobiae*
			Verrucomicrobiales
Anaerostipes caccae (959381)		63.6; 398.9 (total: 462.5)	**Firmicutes**
			*Clostridia*
			Clostridiales
Bacteriodes fragilis (707771)		4.5	**Bacteroidetes**
			*Bacteroidetes*
			Bacteroidales
Bacteriodes thetaiotaomicron (959872)		4.2; 23.1 (total: 27.3)	**Bacteroidetes**
			*Bacteroidetes*
			Bacteroidales
Bacteriodes vulgatus (959871)	6.9	88.9	**Bacteroidetes**
			*Bacteroidetes*
			Bacteroidales
Blautia hydrogenotrophica (959990)		2.8	**Firmicutes**
			*Clostridia*
			Clostridiales
Brevibacillus brevis (960094)		4.4; 8.7 (total:13.1)	**Firmicutes**
			*Bacilli*
			Bacillales
Campylobacter upsaliensis (960299)		2.1; 13.8; 90.6 (total:106.5)	**Protobacteria**
			*Epsilonproteobacteria *
			Campylobacteriales
Citrobacter freundii (960593)		2.1	**Protobacteria**
			*Gammaproteobacteria*
			Enterobacteriales
Clostridium difficile (960739)	4.3		**Firmicutes**
			*Clostridia*
			Clostridiales
Clostridium perfringens (555646)		4.9; 46.5; 290.0 (total: 341.4)	**Firmicutes**
			*Clostridia*
			Clostridiales
Enterococcus gallinarum (961473),		523.4	**Firmicutes**
			*Bacilli*
Enterococcus casseliflavus (961509)			Lactobacillales
Enterococcus faecalis (961474)		4.9; 9.1; 56.5 (total: 70.5)	**Firmicutes**
			*Bacilli*
			Lactobacillales
Enterococcus faecium (961475)		6.8	**Firmicutes**
			*Bacilli*
			Lactobacillales
Plesiomonas shigelloides (964927)		2.7; 3.3; 15.5 (total: 21.5)	**Protobacteria**
			*Gammaproteobacteria*
			Enterobacteriales
Streptococcus agalactiae (449)		3.4	**Firmicutes**
			*Bacilli*
			Lactobacillales
Vibrio parahaemolyticus (85)		9.5; 604.7 (total: 614.2)	**Protobacteria**
			*Gammaproteobacteria*
			Vibrionales

The Actinobacteria phylum and the bacterial orders of Thiotrichales, Selemonadales and Actinomycetales were not detected in the blood of cirrhotic patients (**Fig 1 and S2 Table**). The blood of control subjects had only 2 of 12 bacterial orders, Bacteroidales and Clostridiales (P = 0.0041 for cirrhotic subjects) in 2 subjects out of 9 (**Table 4**).

In the cirrhotic cohort we detected 19 out of 53 bacterial species tested (**Fig 1 and Table 4).** The number of bacteria species was increased in cirrhotic patients compared to control individuals (0.2+/-0.4 vs 3.1+/-2.3; 95% CI: 1.3 to 4.9; P = 0.0030) (**Table 4**). The bacterial DNA quantity was also increased in the blood of cirrhotic subjects compared to control subjects (0.2+/- 1.1 vs 41.8+/-132.1; 95%CI: 6.0 to 77.2; P = 0.0022) (**Table 4**).

There were statistically significant relationships between bacterial DNA quantity and some laboratory parameters (albumin, total bilirubin, INR, platelets, and CRP) (**S3 Table**). No relationship was found between total bacterial DNA and Hgb A1C, AST or ALT (**S3 Table**).

In cirrhotic subjects, the translocated bacteria corresponded to the following phyla: Firmicutes (9 bacterial species), Protobacteria (6 bacterial species), Bacteroides (3 bacterial species), and Verrucomicrobia (1 bacterium specie) (**Fig 1 and Table 4)**.

In our cirrhotic cohort, we detected 8 bacterial species from 7 bacterial orders in a single subject. The 19 bacterial DNA species detected in the 9 cirrhotic subjects included Aeromonas veronii, Aeromonas sobria, Akkermansia muciniphila, Anaerostipes caccae, Bacteriodes fragilis, Bacteriodes thetaiotaomicron, Bacteriodes vulgatus, Blautia hydrogenotrophica, Brevibacillus brevis, Campylobacter upsaliensis, Citrobacter freundii, Clostridium perfringens, Enterococcus gallinarum, Enterococcus casseliflavus, Enterococcus faecalis, Enterococcus faecium, Plesiomonas shigelloides, Streptococcus agalactiae, and Vibrio parahaemolyticus (**Table 4)**. The bacterial DNA detected in two of the 9 control subjects included: Bacteriodes vulgatus in one, and Clostridium difficile in another (the only bacteria detected in a control subject but not in cirrhotic subjects) (**Table 4)**. The bacteria phyla, class, order and species that were not detected in the blood of the cirrhotic or control subjects are shown in **S2 Table.**

### Expression of Nitic Oxide Signaling Gene Array in Blood Inflammatory Cells

We used the RT^2^ Profiler™ PCR Array Human Nitric Oxide Signaling Pathway (Qiagen) to determine whether this pathway is activated in blood inflammatory cells (‘buffy coat’) by the bacterial DNA translocation in the cirrhotic subjects compared to control subjects. This array contains a panel of proprietary controls to monitor DNA contamination (DC) as well as the first strand synthesis (RTC) and real-time PCR efficiency (PPC). The specificity of each gene amplification is guaranteed by the RT^2^ SYBR Green PCR Array System (Qiagen). Many members of this NO signaling pathway gene family were consistently increased compared to control samples. Genes that were expressed greater than 2-fold the control values included CXCL8 (IL-8), KRT1, APOE, CCNA1 (cyclin A1), DUSP-1, IL-10, and GRIND1 (P < 0.05 for all of these genes) (**S4 Table**).

The Spearman’s correlation between the Nitric Oxide Signaling Pathway genes and the bacterial DNA per cirrhotic subject was modest to moderate (KRT1: -0.68, P = 0.044; APOE: 0.64, P = 0.0633; VEGFA: 0.50, P = 0.1705; IL-10: 0.46, P = 0.2128). A comprehensive documentation of the results of the Nitric Oxide Signaling Pathway genes in cirrhotic patients is shown in **S1 Fig and S2 Fig.**

### Expression of NO, Cytokines/Chemokines and NALP-3 in Blood

**Fig 3:**
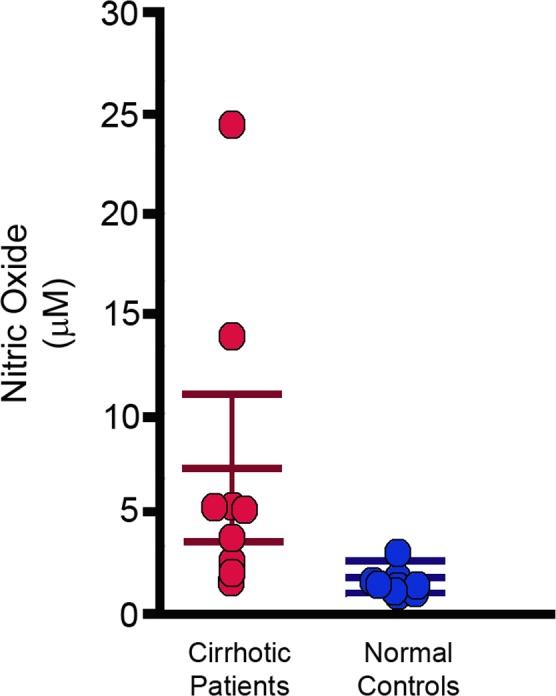
Increased Nitric Oxide in the blood of cirrhotic subjects. Nitric oxide was increased in the blood of cirrhotic subjects compared to control subjects. The P value is indicated using a two-tailed Mann-Whitney U test.

As expected, NO was increased in the blood of cirrhotic subjects compared to control subjects (1.56 +/- 0.58 vs 7.18+/-7.50 μM; 95%CI: 0.71 to 10.53; P = 0.0282) (**Fig 3**). Nitric oxide blood values had a relationship to SVR (-0.71; 95%CI -0.881 to -0.355; P = 0.0011) (**Table 3**) and CO (0.57; 95%CI 0.141 to 0.818; P = 0.0144). The correlation of NO with other hemodynamic parameters was moderate and statistically not significant (data not shown).

Of relevance for the main hypothesis linking bacterial translocation to SVR, at least in part through the production of NO, we found that NO blood levels had a relationship with both bacterial species number (0.64; 95% CI 0.247 to 0.852; P = 0.0041) and bacterial DNA quantity (0.62; 95% CI 0.216 to 0.842; P = 0.0062).

The measurement of 11 cytokines /chemokines potentially induced by bacterial DNA/LPS yielded an increase in the cirrhotic group but these changes from control levels were not statistically significant (except for Rantes that was statistically significantly decreased; 752+/-112 vs 504+/-124; 95% CI 130 to 366; P = %0.0004) (**S5 Table)**. This result was unexpected since Rantes (CCL5) is an important modulator of liver inflammation and its blockade ameliorates experimental liver fibrosis in mice [30].

Similarly, the NALP-3 protein expression, a key modulator of macrophage Inflammasome [6], in the buffy coat of cirrhotic patients’ blood was moderately increased (difference of the means = 29,650; 95% CI -6,453 to 65,753) (**S3 Fig)**.

There was a decrease in the plasma levels of LBP in cirrhotic subjects compared to control subjects (**S4 Fig**) (P = 0.0004).

## Discussion

Our study provides the first correlative analysis of the blood microbiome, inflammatory biomarkers and systemic hemodynamics in a cirrhotic cohort.

In the blood of the cirrhotic cohort we detected 19 of 53 bacterial species tested. The number of bacterial species and the total bacterial DNA were increased in the blood of cirrhotic patients compared to control individuals. In the cirrhotic cohort, the CO increased by 37% while the SVR decreased by 40% compared to control subjects. SVR was inversely correlated to blood bacterial DNA quantity, number of blood bacterial species, and serum NO. These findings strongly support, although do not prove, our premise that the degree of bacterial translocation is mechanistically linked to induction of inflammatory mediators and NO, and that NO and possibly other vasodilators decrease the SVR (while there is a compensatory increase in CO) in cirrhotic patients. Beneficial effects of NOS inhibition have been reported in two patients with life-threatening septic shock in whom conventional therapy had failed to restore blood pressure. NG-monomethyl-L-arginine (L-MMA), an inhibitor of NOS, caused dose-dependent increases in blood pressure and systemic vascular resistance in these patients [11]. Unfortunately, a FDA-approved NOS inhibitor to treat the abnormal SVR in septic or cirrhotic patients is not available.

Although NALP-3 protein expression, a key modulator of macrophage Inflammasome [6], was only moderately increased in the cirrhotic patients’ blood, it is possible that NALP-3 function is modulated in cirrhotic patients by phosphorylation or other post-translational modifications. This is suggested in several of the cirrhotic samples by the presence of more than one NALP-3 protein band in the immunoblot analysis (S3 Fig).

We were able to detect bacteria from 4 out of the 5 phyla (80%) and from 6 out of 8 bacteria classes (75%) in the blood of cirrhotic subjects. We found 9 of 12 bacterial orders (75%) in cirrhotic subjects. The blood of control subjects had only 2 of 12 bacterial orders, Bacteroidales and Clostridiales in 2 out of 9 subjects.

In cirrhotics, the translocated bacteria corresponded to the following phyla: Firmicutes (9 bacterial species), Protobacteria (6 bacterial species), Bacteroidetes (3 bacterial species), and Verrucomicrobia (1 bacterium specie).

We detected some bacterial genera/species that were not detected by Santiago el al [27] in the blood of cirrhotic patients including: Aeromonas veronii, Aeromonas sobria, Anaerostipes caccae, Brevibacillus brevis, Campylobacter upsaliensis, Citrobacter freundii, Plesiomonas shigelloides, and Vibrio parahaemolyticus. Santiago et at [27] detected in the blood of cirrhotic patients the genera Akkermansia, Bacteriodes, Blautia, Clostridium, Enterococcus and Streptococcus but our study further characterized the species as Akkermansia muciniphila, Bacteriodes fragilis, Bacteriodes thetaiotaomicron, Bacteriodes vulgatus, Blautia hydrogenotrophica, Clostridium perfringens, Enterococcus gallinarum, Enterococcus casseliflavus, Enterococcus faecalis, Enterococcus faecium, and Streptococcus agalactiae.

In contrast, the study by Santiago et al [27] was able to identify in the blood of cirrhotic patients bacterial genera not present in our Intestinal Infections Microbial DNA qPCR Array, including: Oscillospora, Phascolarctobacterium, Turicibacter, Acidaminococcus, Desulfovibrio, Eubacterium, Catenibacterium, Phascolarctobacterium, Paraprevotella, Sutterella, Dorea, Alistipes, Megasphaera, Lactobacillus, Megamonas, Lachnobacterium, Veillonella, Bifidobacterium, Prevotella, Parabacteroides, Ruminococcus, Lachnospira, Coprococcus, Roseburia, Succinivibrio, and Escherichia.

The phyla Firmicutes, Bacteroidetes and Proteobacteria represented 92% and 89% of the blood bacterial DNA in the study by Santiago and coworkers [27] and in the present study, respectively. In our study we detected 19/53 bacterial species (36%) and 13/30 genera (43%) tested in the blood microbiome of cirrhotic patients. Santiago et al [27] utilized a more extensive bacterial array, which was less specific to the fecal microbiome; they detected 37/387 bacterial genera (~ 10%) in the blood of cirrhotic patients that were also present in the feces of these patients. Of interest, Santiago et al [27] detected in the blood of cirrhotic patients 246/283 (~ 87%) bacterial genera that were not detected in feces of these patients, suggesting that in their cohort a substantial percentage of the blood microbiome may have originated outside the fecal microbiome.

We suspect that our results measured in the blood (plasma) are an underestimation of the extent of bacterial DNA translocation in cirrhotic patients given that it has been suggested that more bacteria DNA may be present in circulating inflammatory cells than in plasma [31], and that our bacterial DNA test was limited to 53 intestinal pathogenic bacterial species. Pan-bacteria assays that detect a broad range of bacterial species served as positive controls in each subject’s serum. Similarly, negative controls were used in each subject’s plasma to monitor for potential contamination (false positive results). In contrast to the report by Santiago et al [27], we found no bacterial contamination using our Intestinal Infections Microbial DNA qPCR Array method.

Nitric oxide was increased in the blood of cirrhotic subjects compared to control subjects, and NO blood values correlated negatively to SVR and positively to CO. Some genes of the NO signaling array, including CXCL8 (IL-8), KRT1, APOE, CCNA1 (cyclin A1), DUSP-1, IL-10, and GRIND1 were increased in cirrhotic patients. Future investigation of the role bacterial DNA with these NO signaling genes may provide insights into the regulation of this family of genes, and their role in the hyperdynamic circulation of cirrhosis.

In contrast with other studies [25], [27], in our cirrhotic cohort there was a decrease in plasma LBP. Possibly, this reflects differences in cohorts or methodology since LBP binds to LPS and then to monocytes and macrophages, using buffy-coat depleted plasma in our study could have affected the results. These results also suggest that the vast majority of the reported LBP increase in cirrhotic patients may occur within monocytes/macrophages.

Our study suggests that bacterial translocation number and quantity are relevant in the hyperdynamic circulation of cirrhosis, and that they may be valuable parameters for subject’s stratification in clinical studies of portal hypertension. Our study also suggests the potential value of an assessment of the qualitative and quantitative roles of specific species bacterial DNA on the immune responses in normal subjects and cirrhotic patients. As stated by the European Association for the Study of the Liver (EASL) Special Conference 2013 on bacterial infections in cirrhosis, biomarkers of early infection may be useful in early diagnosis and treatment of infections [32], potentially including the biomarkers reported by Santiago et al [27], and by us in this study.

The limitations of our study may include the lack of analysis of the intestinal microbiome and that the sample size was not powered to performed analysis of subgroups. However, analysis of the intestinal microbiome was not pertinent to the primary and secondary aims of the study; and the sample size analysis was appropriately estimated for the primary and secondary goals of the study. A potential bias in finding more substantial changes in SVR and CO than in other cirrhotic populations could have been introduced by enrolling subjects from a single hospital (Veterans Affairs San Diego Healthcare System).

The development of Nitric Oxide modulators with an acceptable risk/benefit may have potential positive effects on the pathological hyperdynamic circulation of cirrhosis.

## Supporting Information

S1 Table Enrollment of control and cirrhotic subjects. (DOCX)

S2 Table Blood bacteria DNA not identified in control and cirrhotic subjects. Bacterial phylum, class, order, and species tested but not detected in the blood of healthy or cirrhotic subjects.
(DOCX)

S3 Table Correlation of Bacterial DNA with laboratory parameters. The correlation of the blood Bacterial DNA with laboratory parameters is shown in the cirrhotic cohort. The P values are indicated for each comparison using the Spearman’s correlation.
(DOCX)

S4 Table NO-related gene levels in cirrhotic subjects. The subjects’ NO-related gene levels are shown for the cirrhotic cohort. The P values and 95% CI are indicated for each comparison to controls.
(DOCX)

S5 Table Cytokine/ chemokine serum levels in control and cirrhotic subjects. The subjects’ cytokine parameters are shown for the control and cirrhotic cohorts. The P values and 95% CI are indicated for each comparison.
(DOCX)

S1 Fig Expression of NO gene array in each cirrhotic subject. The NO gene array expression was determined as described in Methods. Each gene for each cirrhotic subject was compared to the pooled values of control subjects. The magnitude of the gene expression in indicated in the color chart at the bottom of the Figure.
(TIF)

S2 Fig Quantitative expression of Nitic Oxide signaling gene array in each cirrhotic subject. The NO gene array expression was determined as described in Methods. Each gene for each cirrhotic subject was compared to the pooled values of control subjects. Two-fold differences of a gene between a cirrhotic subject and the pooled control subjects were indicated in red and green (increases and decreases, respectively). Genes that remained unchanged are indicated in black. Cirrhotic subjects 1–9 are shown in panels A-I.
(TIF)

S3 Fig Expression of NALP-3 in cirrhotic and control subjects. The NALP-3 protein expression blood inflammatory cell was determined as described in Materials and methods. There were no statistically significant differences between the two cohorts. **A**. An immunoblot of immunoprecipitated NALP-3 corrected for loading by β-actin is shown (C = cirrhotics; H = healthy controls). **B.** Quantification of the immunoblot NALP-3 values for the each subject. There were no statistically significant differences between the two cohorts.
(TIF)

S4 Fig Expression of LBP in cirrhotic and control subjects. The LBP protein expression in blood as determined as described in Materials and methods. There was a decrease in LBP in cirrhotic subjects.
(TIF)

## Abbreviations

BT: Bacterial translocation
MLN: mesenteric lymph nodes
IL: interleukin
TNF: tumor necrosis factor
TX: taxonomic
CR: cardiac rate
SBP: systolic blood pressure
DBP: diastolic blood pressure
MAP: mean arterial pressure
CO: cardiac output
CI: cardiac index
SVR: systemic vascular resistance
SVRI: systemic vascular resistance index
SV: stroke volume
SVI: stroke volume index
VI: velocity index
STR: systolic time ratio
PEP: pre-ejection period
LVET: left ventricular ejection fraction
LCW: left cardiac work
LCWI: left cardiac work index
TFC: thoracic fluid content
ACI: acceleration index
NO: nitric oxide
HVPG: hepatic vein portal gradient
LBP: lipopolysaccharide binding protein

## References

[1] Chojkier M, Fierer J. D-Galactosamine hepatotoxicity is associated with endotoxin sensitivity and mediated by lymphoreticular cells in mice. Gastroenterology. 1985;88(1 Pt 1):115–21. PubMed Central PMCID: PMC3880554. 3880554 10.1016/s0016-5085(85)80142-6

[2] Duffield JS, Forbes SJ, Constandinou CM, Clay S, Partolina M, Vuthoori S, et al Selective depletion of macrophages reveals distinct, opposing roles during liver injury and repair. J ClinInvest. 2005;115(1):56–65. 10.1172/JCI22675 PMC539199 15630444

[3] Petrasek J, Bala S, Csak T, Lippai D, Kodys K, Menashy V, et al IL-1 receptor antagonist ameliorates inflammasome-dependent alcoholic steatohepatitis in mice. The Journal of Clinical Investigation. 2012;122(10):3476–89. 10.1172/JCI60777 22945633 PMC3461900

[4] Ouyang X, Ghani A, Malik A, Wilder T, Colegio OR, Flavell RA, et al Adenosine is required for sustained inflammasome activation via the A2A receptor and the HIF-1α pathway. Nature Communications. 2013. 10.1038/ncomms3909 PMC3895487 24352507

[5] Tsutsui H, Kayagaki N, Kuida K, Nakano H, Hayashi N, Takeda K, et al Caspase-1-Independent, Fas/Fas Ligand–Mediated IL-18 Secretion from Macrophages Causes Acute Liver Injury in Mice. Immunity. 1999;11(3):359–67. 10514014 10.1016/s1074-7613(00)80111-9

[6] Buck M, Solis-Herruzo., Chojkier M. C/EBPβ-Thr217 Phosphorylation Stimulates Macrophage Inflammasome Activation and Liver Injury. Scientific Reports. 2016. 10.1038/srep24268 PMC4828658 27067260

[7] Szabo G, Petrasek J. Inflammasome activation and function in liver disease. Nature Reviews Gastroenterology & Hepatology; 2015 p. 387–400. 26055245 10.1038/nrgastro.2015.94

[8] Nathan C, Xie QW. Regulation of biosynthesis of nitric oxide. J Biol Chem. 1994;269(19):13725–8. 7514592

[9] Ignarro L, Buga G, Wood K, Byrns R, Chaudhuri G. Endothelium-derived relaxing factor produced and released from artery and vein is nitric oxide. PNAS. 1987;84(24):9265–9. PubMed Central PMCID: PMCPMC299734. 2827174 10.1073/pnas.84.24.9265 PMC299734

[10] Bunnell E, Lynn M, Habet K, Neumann A, Perdomo C, Friedhoff L, et al A lipid A analog, E5531, blocks the endotoxin response in human volunteers with experimental endotoxemia. Critical Care Medicine. 2000;28(8):2713–20. PubMed Central PMCID: PMC10966240. 10966240 10.1097/00003246-200008000-00005

[11] Petros A, Bennet D, Vallance P. Effect of nitric oxide synthase inhibitors on hypotension in patients with septic shock. The Lancet. 1991;338:1557–91. 10.1016/0140-6736(91)92376-d 1720856

[12] Nava E, Palmer RM, Moncada S. Inhibition of nitric oxide synthesis in septic shock: how much is beneficial? Lancet. 1991;338:1555–7. 1683974 10.1016/0140-6736(91)92375-c

[13] Whittle B, Moncada S. Nitric oxide: the elusive mediator of the hyperdynamic circulation of cirrhosis? Hepatology. 1992;16(4):1089–92. PubMed Central PMCID: PMC1398490. 1398490 10.1002/hep.1840160437

[14] Bomzon A, Blendis LM. The Nitric Oxide Hypothesis and the Hyperdynamic Circulation in Cirrhosis. Hepatology. 1994;20(5):1343–50. PubMed Central PMCID: PMC7927270. 7927270

[15] Iwakiri Y, Groszmann RJ. The hyperdynamic circulation of chronic liver diseases: From the patient to the molecule. Hepatology. 2006;43(S1):S121–S31. 16447289 10.1002/hep.20993

[16] Beutler B. TLR4 as the mammalian endotoxin sensor. Curr Top Microbiol Immunol. 2002;270:109–20. 12467247 10.1007/978-3-642-59430-4_7

[17] Hemmi H, Takeuchi O, Kawai T, Kaisho T, Sato S, Sanjo H, et al A Toll-like receptor recognizes bacterial DNA. Nature. 2000;408:740–5. 10.1038/35047123 11130078

[18] Wiest R, Lawson M, Geuking M. Pathological bacterial translocation in liver cirrhosis. Journal of Hepatology. 2012;60(1):197–209. 10.1016/j.jhep.2013.07.044 23993913

[19] Villanueva C, Albillos A, Genesca J, Abraldes JG, Calleja JL, Aracil C, et al Development of hyperdynamic circulation and response to β-blockers in compensated cirrhosis with portal hypertension. Hepatology. 2015;63(1):197–206. 10.1002/hep.28264 26422126

[20] Bosch J, Berzigotti A, Garcia-Pagan JC. Portal hypertension and gastrointestinal bleeding. Semi Liver Dis. 2008;28(1):3–25. 10.1055/s-2008-1040318 18293274

[21] Abraldes JG, Tarantino I, Turnes J, Garcia-Pagan JC, Rodes J, Bosch J. Hemodynamic response to pharmacological treatment of portal hypertension and long-term prognosis of cirrhosis. Hepatology. 2003;37(4):902–8. 10.1053/jhep.2003.50133 12668985

[22] Bellot P, Garcia-Pagan JC, Frances R, Abraldes JG, Navasa M, Perez-Mateo M, et al Bacterial DNA translocation is associated with systemic circulatory abnormalities and intrahepatic endothelial dysfunction in patients with cirrhosis. Hepatology. 2010;52(6):2044–52. 10.1002/hep.23918 20979050

[23] Frances R, Rodriguez E, Munoz C, Zapater P, De la ML, Ndongo M, et al Intracellular cytokine expression in peritoneal monocyte/macrophages obtained from patients with cirrhosis and presence of bacterial DNA. Eur J Gastroenterol Hepatol. 2005;17(1):45–51. 15647640 10.1097/00042737-200501000-00010

[24] Frances R, Zapater P, Gonzalez-Navajas JM, Munoz C, Cano R, Moreu R, et al Bacterial DNA in patients with cirrhosis and noninfected ascites mimics the soluble immune response established in patients with spontaneous bacterial peritonitis. Hepatology. 2008;47(3):978–85. 10.1002/hep.22083 18306221

[25] Albillos A, de la Hera A, Gonzalez M, Moya J-L, Calleja J-L, Monserrat J, et al Increased Lipopolysaccharide Binding Protein in Cirrhotic Patients With Marked Immune and Hemodynamic Derangement. Hepatology. 2003;37(1):208–17. 10.1053/jhep.2003.50038 12500206

[26] Albillos A, Lario M, Alvarez-Mon M. Cirrhosis-associated immune dysfunction: Distinctive features and clinical relevance. Journal of Hepatology. 2014;61:1385–96. 10.1016/j.jhep.2014.08.010 25135860

[27] Santiago A, Pozuelo M, Poca M, Gely C, Nieto JC, Torras X, et al Alteration of the serum microbiome composition in cirrhotic patients with ascites. Scientific Reports 2016;6(25001). 10.1038/srep25001 PMC4845009 27112233

[28] Tang WHW, Tong W. Measuring impedance in congestive heart failure: Current options and clinical applications. American Heart. 2008;157(3):402–11. 10.1016/j.ahj.2008.10.016 PMC3058607 19249408

[29] Buck M, Garcia-Tsao G, Groszmann RJ, Stalling C, Grace ND, Burroughs AK, et al Novel inflammatory biomarkers of portal pressure in compensated cirrhosis patients. Hepatology. 2014;59(3):1052–9. 10.1002/hep.26755 24115225

[30] Berres M-L, Koenen RR, Rueland A, Zaldivar MM, Heinrichs D, Sahin H, et al Antagonism of the chemokine Ccl5 ameliorates experimental liver fibrosis in mice. The Journal of Clinical Investigation. 2010;120(11):4129–40. 10.1172/JCI41732 20978355 PMC2964968

[31] Paisse S, Valle C, Servant F, Courtney M, Burcelin R, Amar J, et al Comprehensive description of blood microbiome from healthy donors assessed by 16S targeted metagenomic sequencing. Transfusion. 2016. 10.1111/trf.13477 26865079

[32] Jalan R, Fernandez J, Wiest R, Schnabl B, Moreau R, Angeli P, et al Bacterial infections in cirrhosis: A position statement based on the EASL Special Conference 2013. Journal of Hepatology. 2014;60(6):1310–24. 10.1016/j.jhep.2014.01.024 24530646

