# Supplementary material for: Blood Microbiome Quantity and the Hyperdynamic Circulation in Decompensated Cirrhotic Patients
Source: PLoS One. 2017 Feb 1;12(2):e0169310. doi: 10.1371/journal.pone.0169310 (PMC5287452; doi:10.1371/journal.pone.0169310)
Supplement: S1 Table — (DOCX) [file pone.0169310.s001.docx]

Assessed for eligibility (n-24)

CIRRHOTIC

Control

Assessed for eligibility (n-17)

CONTROL

Control

## Enrollment

Excluded (n=8)

♦  Not meeting inclusion criteria (n=5)

♦  Declined to participate (n=1)

♦  Other reasons (n=2)

Excluded (n=13)

♦  Not meeting inclusion criteria (n=10)

♦  Declined to participate (n=2)

♦  Other reasons (n=1)

Allocated to control group (n=9)

## Allocation

Allocated to cirrhotic group (n= 9)

## Analysis

Analyzed (n=9)
♦ Excluded from analysis (n=0)

Analyzed (n=9)
♦ Excluded from analysis (n=0)
