# Supplementary material for: Blood Microbiome Quantity and the Hyperdynamic Circulation in Decompensated Cirrhotic Patients
Source: PLoS One. 2017 Feb 1;12(2):e0169310. doi: 10.1371/journal.pone.0169310 (PMC5287452; doi:10.1371/journal.pone.0169310)
Supplement: S2 Table — Bacterial phylum, class, order, and species tested but not detected in the blood of healthy or cirrhotic subjects. (DOCX) [file pone.0169310.s002.docx]

| **Blood Bacteria**  (Taxonomic #) | **Phylum**  *Class*  Order | |
| --- | --- | --- |
| Acidaminococcus fermentans  (958723) | **Firmicutes**  *Negativicutes*  Selenomonadales | |
| Aeromonas enteropelogenes (959039),  Aeromonas hydrophilia (77),  Aeromonas media  (959046),  Aeromonas punctata (80) | **Protobacteria**  *Gammaproteobacteria*  Aeromonadales | |
| Anaerococus lactolyticus  (959360) | **Firmicutes**  *Clostridia*  [Clostridiales](http://www.itis.gov/servlet/SingleRpt/SingleRpt?search_topic=TSN&search_value=956209) | |
| Anaerococcus colihominis  ( 959383) | **Firmicutes**  *Clostridia*  [Clostridiales](http://www.itis.gov/servlet/SingleRpt/SingleRpt?search_topic=TSN&search_value=956209) | |
| Bacillus anthracis  (959822),  Bacillus cereus  (959821) | **Firmicutes**  *Bacilli*  Bacillales | |
| Butyricicoccus pullicaecorum  (960237) | **Firmicutes**  *Clostridia*  [Clostridiales](http://www.itis.gov/servlet/SingleRpt/SingleRpt?search_topic=TSN&search_value=956209) | |
| Campylobacter coli  (960312),  Campylobacter jejuni  (958568),  Campylobacter lari  (958564),  Campylobacter subantarticus  (960311), | [**Proteobacteria**](http://www.itis.gov/servlet/SingleRpt/SingleRpt?search_topic=TSN&search_value=956120)  *Epsilonproteobacteria*  [Campylobacterales](http://www.itis.gov/servlet/SingleRpt/SingleRpt?search_topic=TSN&search_value=956346) | |
| Campylobacter fetus  (958566) | [**Proteobacteria**](http://www.itis.gov/servlet/SingleRpt/SingleRpt?search_topic=TSN&search_value=956120)  *Epsilonproteobacteria*  [Campylobacterales](http://www.itis.gov/servlet/SingleRpt/SingleRpt?search_topic=TSN&search_value=956346) | |
| Clostridium septicum  (960764) | **Firmicutes**  *Clostridia*  [Clostridiales](http://www.itis.gov/servlet/SingleRpt/SingleRpt?search_topic=TSN&search_value=956209) | |
| Enterococcus italicus  (961495) | **Firmicutes**  *Bacilli*  Lactobacillales | |
| Enterobacter cloacae  (248),  Klebsiella oxytoca  (962549) | **Protobacteria**  *Gammaproteobacteria*  Enterobacteriales | |
| Francisella novicida  (968481),  Francisella tularensis  (958578) | **Protobacteria**  *Gammaproteobacteria*  [Thiotrichales](http://www.itis.gov/servlet/SingleRpt/SingleRpt?search_topic=TSN&search_value=956282) | |
| Helicobacter cinaedi  (962226 ) | [**Proteobacteria**](http://www.itis.gov/servlet/SingleRpt/SingleRpt?search_topic=TSN&search_value=956120)  *Epsilonproteobacteria*  [Campylobacterales](http://www.itis.gov/servlet/SingleRpt/SingleRpt?search_topic=TSN&search_value=956346) | |
| Helicobacter fennelliae  (962227) | [**Proteobacteria**](http://www.itis.gov/servlet/SingleRpt/SingleRpt?search_topic=TSN&search_value=956120)  *Epsilonproteobacteria*  [Campylobacterales](http://www.itis.gov/servlet/SingleRpt/SingleRpt?search_topic=TSN&search_value=956346) | |
| Listeria monocytogenes  (963001) | **Firmicutes**  *Bacilli*  Bacillales | |
| Morganella morganii  (958592) | **Protobacteria**  *Gammaproteobacteria*  Enterobacteriales | |
| Mycobacterium avium  (958595) | **Actinobacteria**   \| [*Actinobacteridae*](http://www.itis.gov/servlet/SingleRpt/SingleRpt?search_topic=TSN&search_value=956179) \| \| --- \| \| [Actinomycetales](http://www.itis.gov/servlet/SingleRpt/SingleRpt?search_topic=TSN&search_value=465) \| | |
| Mycobacterium intracellulare  (963819) | **Actinobacteria**   \| [*Actinobacteridae*](http://www.itis.gov/servlet/SingleRpt/SingleRpt?search_topic=TSN&search_value=956179) \| \| --- \| \| [Actinomycetales](http://www.itis.gov/servlet/SingleRpt/SingleRpt?search_topic=TSN&search_value=465) \| | |
| Shigella dysenteriae  (966038) | | **Protobacteria**  *Gammaproteobacteria*  Enterobacteriales |
| Streptococcus pyogenes  (966482) | | **Firmicutes**  *Bacilli*  Bacillales |
| Streptococcus suis  (966471) | | **Firmicutes**  *Bacilli*  Lactobacillales |
| Vibrio cholera  (967647) | | **Protobacteria**  *Gammaproteobacteria*  Vibrionales |
| Vibrio vulnificus  (967565) | | **Protobacteria**  *Gammaproteobacteria*  Vibrionales |
| Yersinia enterocolitica  (958655) | | **Protobacteria**  *Gammaproteobacteria*  Enterobacteriales |
| Yersinia pestis  (967822),  Yersinia pseudotuberculosis  (967821) | | **Protobacteria**  *Gammaproteobacteria*  Enterobacteriales |
