# Supplementary material for: Blood Microbiome Quantity and the Hyperdynamic Circulation in Decompensated Cirrhotic Patients
Source: PLoS One. 2017 Feb 1;12(2):e0169310. doi: 10.1371/journal.pone.0169310 (PMC5287452; doi:10.1371/journal.pone.0169310)
Supplement: S3 Table — The correlation of the blood Bacterial DNA with laboratory parameters is shown in the cirrhotic cohort. The P values are indicated for each comparison using the Spearman’s correlation. (DOCX) [file pone.0169310.s003.docx]

| **Parameter** | **Spearman’s**  **Correlation** | **P value** (95% CI) ; **R^2^** |
| --- | --- | --- |
| Albumin (g/dL) | -0.693 | 0.0014 (-0.876 to -0.335); 0.185 |
| Total Bilirubin (mg/dL) | 0.633 | 0.0047 (-0.848 to -0.236); 0.072 |
| INR (ratio) | 0.631 | 0.0049 (0.233 to 0.848); 0.172 |
| Platelets (x 10^3^/μL) | -0.617 | 0.0063 (-0.841 to -0.211); 0.281 |
| CRP (mg/dL) | 0.528 | 0.0242 (0.082 to 0.798); 0.098 |
| Hgb A1c (%) | -0.282 | 0.2565 (-0.661 to 0.212); |
| AST (IU/ml) | 0.339 | 0.1675 (-0.151 to 0.695); 0.055 |
| ALT (IU/ml) | -0.003 | 0.9894 (-0.469 to 0.464); 0.196 |
