# Supplementary material for: Blood Microbiome Quantity and the Hyperdynamic Circulation in Decompensated Cirrhotic Patients
Source: PLoS One. 2017 Feb 1;12(2):e0169310. doi: 10.1371/journal.pone.0169310 (PMC5287452; doi:10.1371/journal.pone.0169310)
Supplement: S4 Table — The subjects’ NO-related gene levels are shown for the cirrhotic cohort. The P values and 95% CI are indicated for each comparison to controls. (DOCX) [file pone.0169310.s004.docx]

| **Parameter** | **Cirrhotic** (mean +/- SD ) | **P value (95% CI)** |
| --- | --- | --- |
| CXCL8 | 10.7+/-11.1 | 0.0185 (1.8 to 17.5) |
| KRT1 | 10.2+/-10.2 | 0.0156 (2.0 to 16.4) |
| APOE | 3.3+/-2.1 | 0.0047 (0.8 to 3.8) |
| CCNA1 | 3.3+/-2.2 | 0.0064 (0.7 to 3.9) |
| DUSP1 | 2.7+/-1.9 | 0.0163 (0.4 to 3.0) |
| IL-10 | 2.6+/-1.1 | 0.0005 (0.8 to 2.4) |
| GRIND1 | 2.5+/-1.4 | 0.0054 (0.5 to 2.5) |
