# Supplementary material for: Blood Microbiome Quantity and the Hyperdynamic Circulation in Decompensated Cirrhotic Patients
Source: PLoS One. 2017 Feb 1;12(2):e0169310. doi: 10.1371/journal.pone.0169310 (PMC5287452; doi:10.1371/journal.pone.0169310)
Supplement: S5 Table — The subjects’ cytokine parameters are shown for the control and cirrhotic cohorts. The P values and 95% CI are indicated for each comparison. (DOCX) [file pone.0169310.s005.docx]

| **Parameter** (pg/mL) | **Control** (mean +/- SD ) | **Cirrhotic** (mean +/- SD ) | **P value** (95 % CI) |
| --- | --- | --- | --- |
| TNFα | 58+/-54 | 293+/-440 | 0.1308 (-548 to 78) |
| IL-1β | 72+/-124 | 151+/-206 | 0.3418 (-249 to 91) |
| IL-6 | 13+/-18 | 129+/-303 | 0.2945 (-330 to 98) |
| IL-8 | 45+/-33 | 158+/-217 | 0.1672 (-268 to 42) |
| IL-12 | 2+/-7 | 15+/-28 | 0.2260 (-33 to 7) |
| IL-17 | 94+/-136 | 219+/-321 | 0.3268 (-371 to 121) |
| Mcp-1 | 30+/-10 | 104+/-142 | 0.1624 (-175 to 27) |
| Mip-1α | 82+/-104 | 180+/-275 | 0.3575 (-306 to 110) |
| Mdc | 24+/-12 | 35+/-15 | 0.0816 (-25 to 3) |
| Eotaxin | 180+/-175 | 355+/-293 | 0.1655 (-416 to 66) |
| Rantes | 752+/-112 | 504+/-124 | 0.0004 (130 to 366) |
