# Supplementary figures and images for: Blood Microbiome Quantity and the Hyperdynamic Circulation in Decompensated Cirrhotic Patients
Source: PLoS One. 2017 Feb 1;12(2):e0169310. doi: 10.1371/journal.pone.0169310 (PMC5287452; doi:10.1371/journal.pone.0169310)

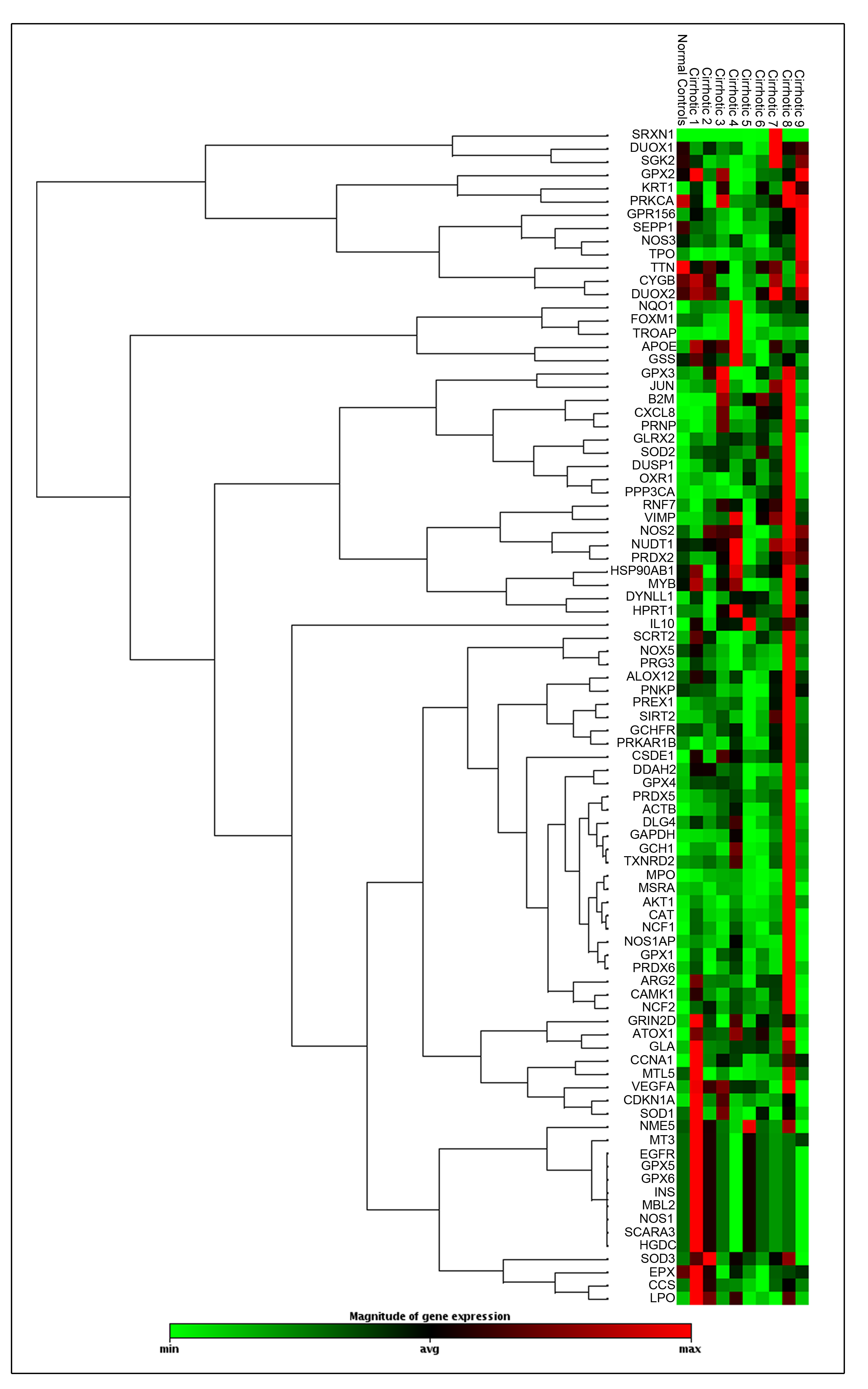

Supplement: S1 Fig — The NO gene array expression was determined as described in Methods. Each gene for each cirrhotic subject was compared to the pooled values of control subjects. The magnitude of the gene expression in indicated in the color chart at the bottom of the Figure. (TIF) [file pone.0169310.s006.tif]

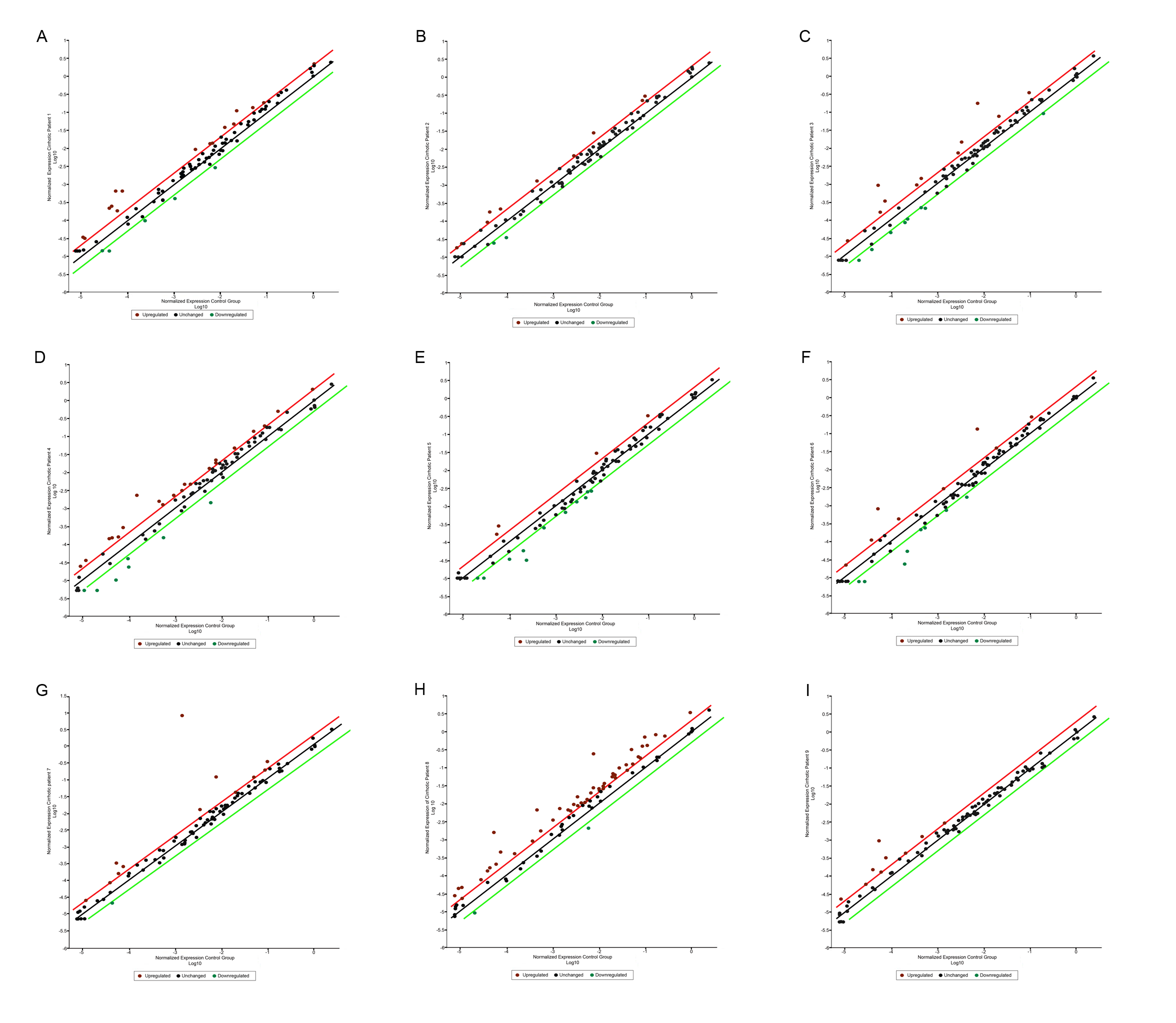

Supplement: S2 Fig — The NO gene array expression was determined as described in Methods. Each gene for each cirrhotic subject was compared to the pooled values of control subjects. Two-fold differences of a gene between a cirrhotic subject and the pooled control subjects were indicated in red and green (increases and decreases, respectively). Genes that remained unchanged are indicated in black. Cirrhotic subjects 1–9 are shown in panels A-I. (TIF) [file pone.0169310.s007.tif]

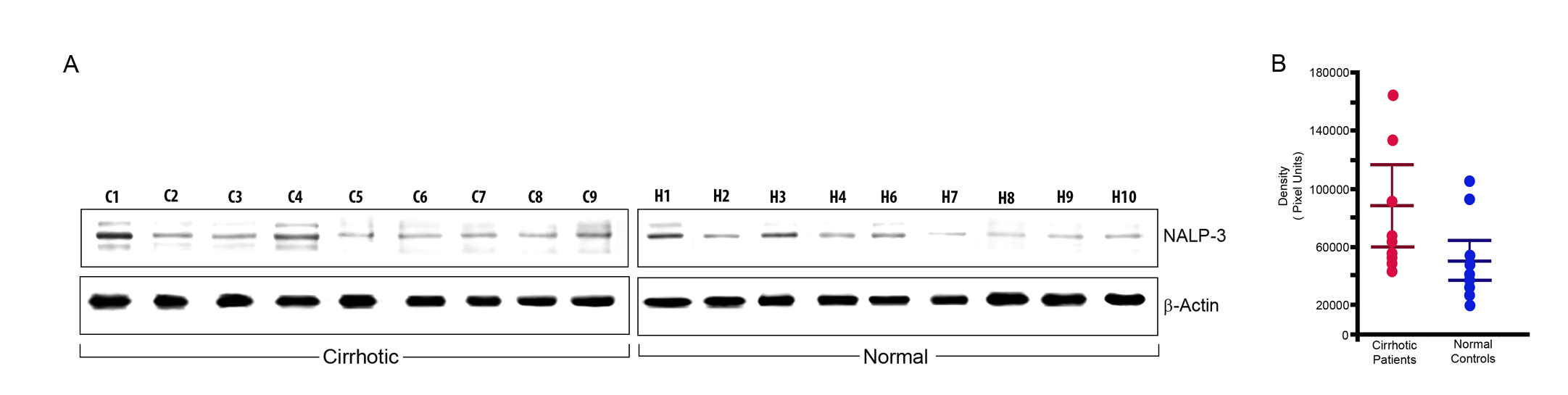

Supplement: S3 Fig — The NALP-3 protein expression blood inflammatory cell was determined as described in Materials and methods. There were no statistically significant differences between the two cohorts. A. An immunoblot of immunoprecipitated NALP-3 corrected for loading by β-actin is shown (C = cirrhotics; H = healthy controls). B. Quantification of the immunoblot NALP-3 values for the each subject. There were no statistically significant differences between the two cohorts. (TIF) [file pone.0169310.s008.tif]

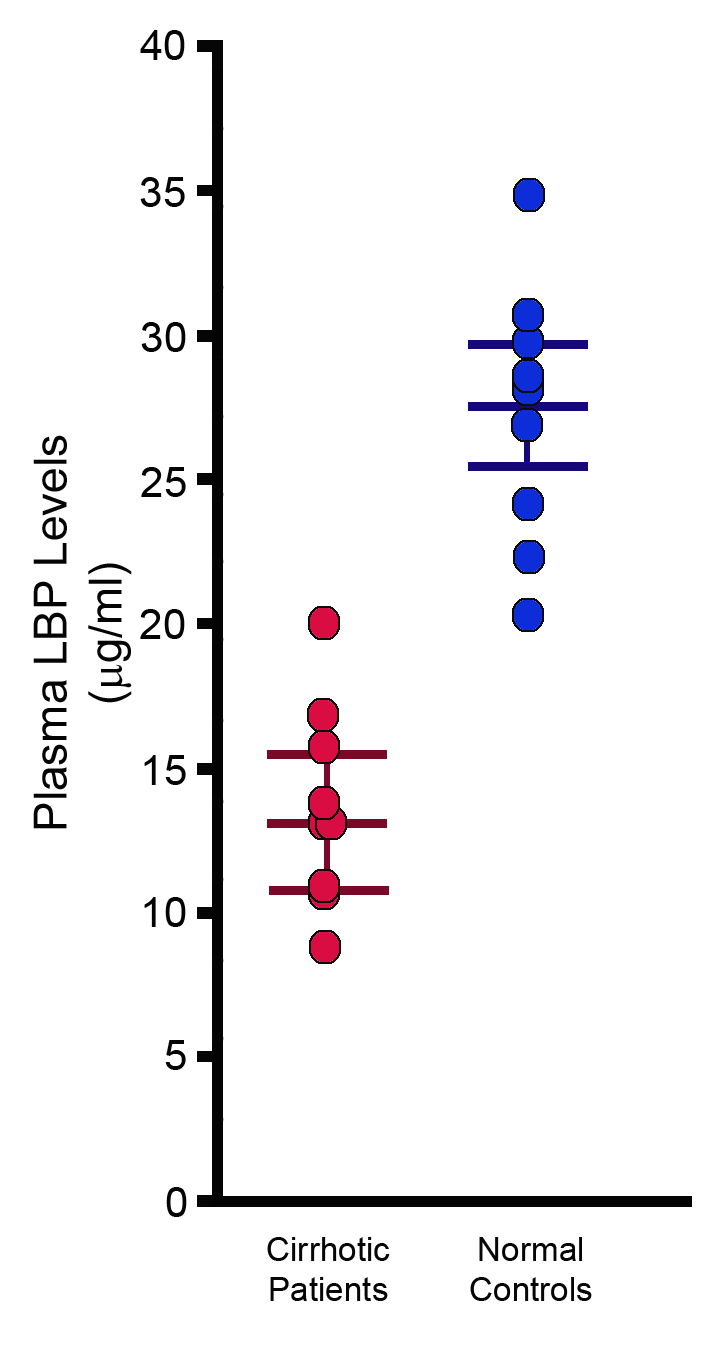

Supplement: S4 Fig — The LBP protein expression in blood as determined as described in Materials and methods. There was a decrease in LBP in cirrhotic subjects. (TIF) [file pone.0169310.s009.tif]
